# Exploring the diversity and evolutionary strategies of prophages in Hyphomicrobiales, comparing animal-associated with non-animal-associated bacteria

**DOI:** 10.1186/s12866-024-03315-3

**Published:** 2024-05-09

**Authors:** Jonathan Gonçalves-Oliveira, Tyler Pattenden, Yaarit Nachum-Biala, Keyla Carstens M. de Sousa, Lindi Wahl, Shimon Harrus

**Affiliations:** 1 Koret School of Veterinary Medicine, The Hebrew University of Jerusalem, Rehovot, Israel; 2 School of Management, Economics and Mathematics, King’s University College, Western University, London, ON Canada; 3 Department of Applied Mathematics, Western University, London, ON Canada

**Keywords:** Hyphomicrobiales, *Brucella*, *Bartonella*, Bacteriophages, Genetic repertoire, Prophage genes, Defense systems

## Abstract

The Hyphomicrobiales bacterial order (previously Rhizobiales) exhibits a wide range of lifestyle characteristics, including free-living, plant-association, nitrogen-fixing, and association with animals (*Bartonella* and *Brucella*). This study explores the diversity and evolutionary strategies of bacteriophages within the Hyphomicrobiales order, comparing animal-associated (AAB) with non-animal-associated bacteria (NAAB). We curated 560 high-quality complete genomes of 58 genera from this order and used the PHASTER server for prophage annotation and classification. For 19 genera with representative genomes, we curated 96 genomes and used the Defense-Finder server to summarize the type of anti-phage systems (APS) found in this order. We analyzed the genetic repertoire and length distributions of prophages, estimating evolutionary rates and comparing intact, questionable, and incomplete prophages in both groups. Analyses of best-fit parameters and bootstrap sensitivity were used to understand the evolutionary processes driving prophage gene content. A total of 1860 prophages distributed in Hyphomicrobiales were found, 695 in AAB and 1165 in the NAAB genera. The results revealed a similar number of prophages per genome in AAB and NAAB and a similar length distribution, suggesting shared mechanisms of genetic acquisition of prophage genes. Changes in the frequency of specific gene classes were observed between incomplete and intact prophages, indicating preferential loss or enrichment in both groups. The analysis of best-fit parameters and bootstrap sensitivity tests indicated a higher selection coefficient, induction rate, and turnover in NAAB genomes. We found 68 types of APS in Hyphomicrobiales; restriction modification (RM) and abortive infection (Abi) were the most frequent APS found for all Hyphomicrobiales, and within the AAB group. This classification of APS showed that NAAB genomes have a greater diversity of defense systems compared to AAB, which could be related to the higher rates of prophage induction and turnover in the latter group. Our study provides insights into the distributions of both prophages and APS in Hyphomicrobiales genomes, demonstrating that NAAB carry more defense systems against phages, while AAB show increased prophage stability and an increased number of incomplete prophages. These results suggest a greater role for domesticated prophages within animal-associated bacteria in Hyphomicrobiales.

**Supplementary Information:**

The online version contains supplementary material available at 10.1186/s12866-024-03315-3.

## Introduction

Bacteriophages (or phages) are viruses that infect bacteria and comprise the most abundant group of organisms on the planet. Recently, lytic phages have been used as alternatives to antibacterials due to the global increase in antimicrobial resistance [1], underscoring the need for a deeper understanding of these organisms. Even though, phage diversity remains poorly explored and largely underestimated [2]. The life cycle of phages includes either a lytic or a lysogenic phase. Phages can be triggered into the lytic phase, in which they destroy the host cell and produce new phage particles (virions). In the lysogenic phase, where the phage DNA is integrated into the bacterial genome as a prophage, the host cell remains intact and the phage replicates through host cell fission or horizontal gene transfer (HGT). Thus, prophages can be a source of genetic variation, including new genes and eventually new functions, enhancing bacteria survival and resistance [3]. On the other hand, the arms race between bacteria and phages involves co-evolutionary pressure, enabling the emergence of several bacterial antiviral systems against phages and new phage strategies to overcome the latter [4]. As part of this arms race, the process of prophage sequence degradation can be seen as a bacterial strategy to ensure that intact prophages are not activated and thus do not initiate the lytic cycle in the host bacterium [5].

The bacterial order Hyphomicrobiales (previously known as Rhizobiales) shows diverse lifestyle traits, ranging from free-living bacteria to nitrogen-fixing in legumes, as well as new genera such as *Bartonella* and *Brucella* that are animal-associated [6]. Both animal-associated bacterial (AAB) genera are considered slow-growing bacteria (with a generation time of more than 2.5 h) and have a short genome size that varies from 3.3 Mb in *Brucella* to about 1.5–2.0 Mb in *Bartonella* [7]. This adaptive radiation was influenced by the loss and acquisition of genes over time, mainly the gain of *VirB* genes that encode the type 4 secretion systems (T4SS) in the AAB genera [8], which have played an important role in the genetic evolution of *Bartonella* and *Brucella* [9–11].

Here, we explored the diversity of the prophages within the genomes of the Hyphomicrobiales to shed light on their evolution within these genomes. Our approach allows us to elucidate the integration process of these mobile genetic elements and understand the evolutionary strategies used by bacterial lineages against these phages. Additionally, we characterized anti-phage systems (APS) and the functionality, length and gene repertoire of prophages and explored differences in the evolutionary forces acting on temperate phages, contrasting prophages from AAB (*Bartonella* and *Brucella*) with those from non-animal-associated bacteria (NAAB).

## Methods

### Data mining

We curated the Hyphomicrobiales genomes that are available in the Bacterial and Viral Bioinformatics Resource Center (BV-BRC/ PATRIC database) [12]. The evaluation of the quality of the genomes was done according to the parameters of fine consistency and high coverage as provided by BV-BRC, such that only complete genomes of good quality (coverage > 90%) and fine consistency (≥ 95%) were analyzed; duplications or unclassified data were removed (Supplementary Fig. 1). Out of 11,511 genomes in the order Hyphomicrobiales that were deposited in BV-BRC (https://www.bv-brc.org/ accessed in October 1, 2022), only 560 genomes with a total of 895 contigs met our stringent quality criteria and were included in subsequent analyses (See Supplementary Table 1). The lifestyle and evolutionary traits of Hyphomicrobiales were identified based on the classification of Wang et al. [6].

**Fig. 1:**
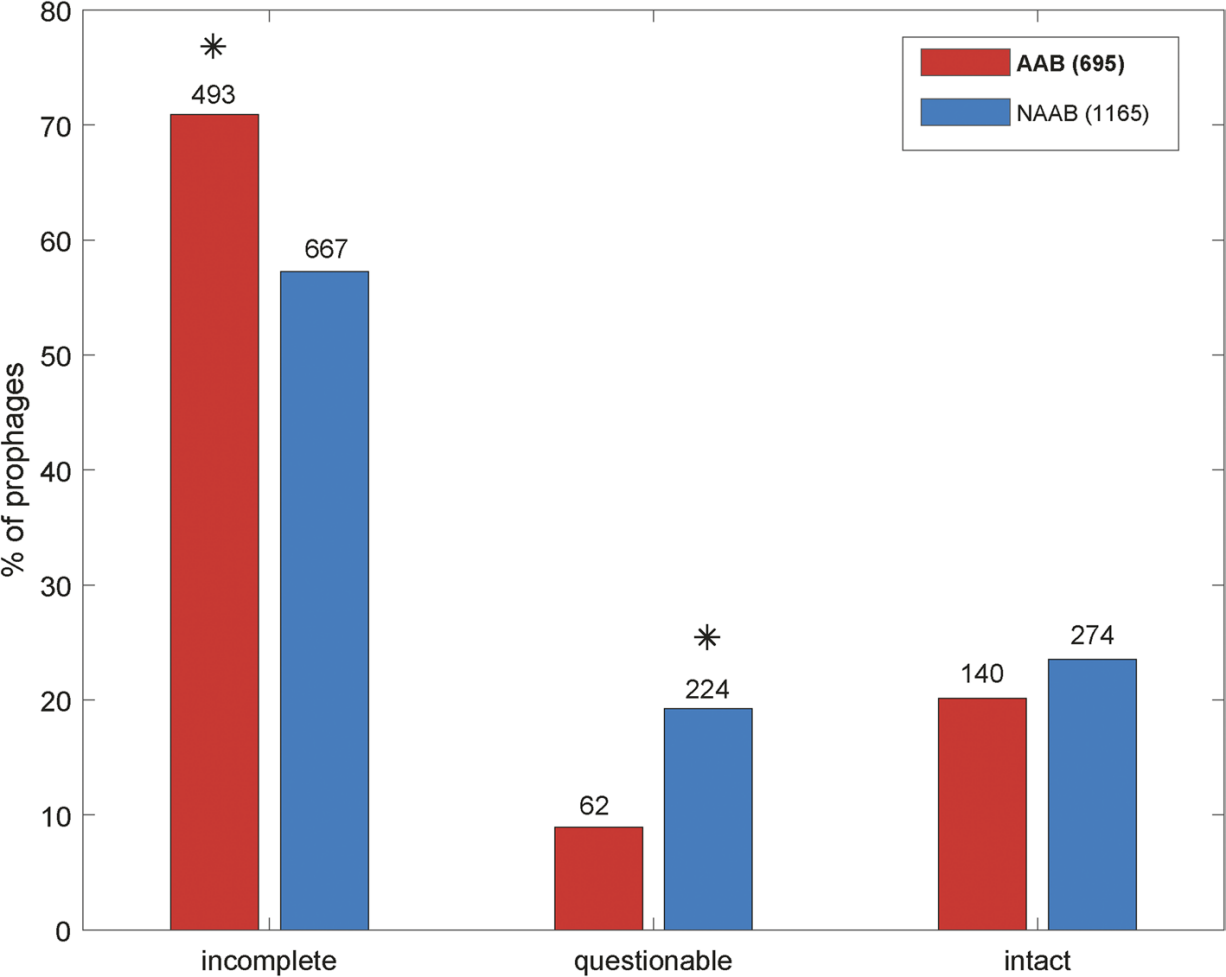
Percentage of prophages in host genomes and the PHASTER classification: (red) animal-associated bacteria (AAB) and (blue) non-animal-associated bacteria (NAAB) – other genera of Hyphomicrobiales. The numbers on the red and blue bars indicate the numbers of prophages of AAB and NAAB genomes in each PHASTER classifications. The stars indicate significant differences between the two groups of genomes at the 5% significance level, two-sided test, Bonferroni correction for three comparisons in each case

Additionally, to classify APS, we followed the methodology proposed by Wang et al. (6, illustrated in Fig. 1). The primary genera associated with specific lifestyles, such as *Hyphomicrobium*, *Methylobacterium*, *Bradyrhizobium*, *Devosia*, *Sinorhizobium*, *Agrobacterium*, *Rhizobium*, *Mesorhizobium*, *Bartonella*, and *Brucella*, were selected for our analysis. These genera were then curated based on the criterion of ‘representative’ genomes available in BV-BRC (accessed on November 5, 2023, at https://www.bv-brc.org/). Consequently, the genomes were reclassified into 19 distinct genera, as detailed in Supplementary Table 2. Furthermore, we employed a dataset of 96 genomes comprising 265 contigs to classify the APS utilizing the Defense Finder server (https://defense-finder.mdmparis-lab.com/), which automatically identifies known antiviral systems in prokaryotic genomes [13]. All 265 contigs underwent analysis, and the results summarized the types of defense systems present in these genomes, enabling the characterization of APS frequency within the Hyphomicrobiales order.

### Prophage analysis

The genomes were submitted to the PHASTER server to annotate phage regions and to classify prophage sequences as intact, questionable, or incomplete [14]. These ‘completeness classes’ are determined by PHASTER using several factors, including prophage length, the number of phage-like genes identified within the sequence, and their identity [15]. The three categories reflect the certainty of the PHASTER algorithm in identifying a gene region as a full, functional prophage. Thus ‘intact’ prophages are considered very likely to be full prophages, whereas ‘incomplete’ prophages are likely partially degraded prophage remnants. Prophage sequences with scores between these two extremes are classified as ‘questionable’. This analysis allowed us to compute the number of prophages identified per genome, the fraction of these prophages in each completeness class, and the length distribution of prophage sequences, all of which were compared between prophages found in AAB and NAAB.

The genetic repertoire of prophages in these three completeness classes was also compared, as described in Khan et al. [16]. In brief, for each coding sequence within an identified prophage region, PHASTER annotates BLAST hits for that sequence, as described in Zhou et al. [15]. We recorded instances within these annotations of the following 13 classes of phage genes: injection (*injn*), plate (*plat*), flippase (*flip*), capsid (*caps*), terminase (*term*), head (*head*), lysin (*lysn*), portal (*port*), lysis (*lyss*), integrase (*intg*), tail (*tail*), transposase (*tran*), protease (*prot*). For these 13 phage gene classes, we counted the number of prophages identified as containing at least one gene of that class. We noted that some gene classes may not be observed in intact prophages in this dataset. This does not imply that these genes are necessarily absent in these prophages, but could be an observational bias based on the available annotated gene sequences used for BLAST comparisons, BLAST parameter settings, or simply small numbers. When a gene class was not observed in intact prophages, this class was excluded from further analysis.

We further examined the gene repertoire annotations based on whether the prophage sequence was classified as “intact”, “questionable” or “incomplete”. This allowed us to calculate the percent change in the frequency of specific classes of prophage genes in degraded prophage sequences, as compared to intact prophages [16]. The percentage change in gene frequency indicates whether certain genes are observed in incomplete prophages more or less frequently than expected, based on their frequency in intact prophages. Specifically, positive values indicate that a particular gene is enriched in incomplete prophages, or preferentially retained as the prophage sequence degrades. In contrast, negative values indicate that this gene group is lost more quickly than others. To evaluate the statistical significance of differences in percentage change, we randomly assigned the same total number of genes to one of the three completeness classes, preserving the proportion of genes assigned to each class [17]. We then computed the percentage change in gene frequency for these bootstrapped, randomly allocated data, and repeated this procedure 10,000 times to assess whether an observed change was significant at the 5% level, including Bonferroni corrections. Two categories of statistical significance were defined: (a) genes that are preferentially lost in incomplete prophages; and (b) genes enriched in incomplete prophages.

The prophage length distributions were also fit to a mathematical model to quantify any differences in the evolutionary rates affecting prophages in each bacterial category (AAB or NAAB). This model was developed to describe the prophage length distribution in two large prophage datasets [16] and later adapted to quantify differences in prophage content across classes of bacteria [17], as we do here. In this model, a partial differential equation describes changes to the length distribution of prophages over time:

$$\frac{\partial Q(x,t)}{\partial t}= \alpha f\left(x\right)+ \frac{\partial }{\partial x}\left[rD\ x\ Q\left(x,t\right)\right]+\left[{r}_{s }S\left(x\right)-{r}_{I}I\left(x\right)\right]Q\left(x,t\right)-\delta \left(t\right)Q(x,t)$$

In the above equation, Q(x, t) is the density of prophages of length x (kb) at time t. The parameter α represents the rate of lysogeny, and f(x) is the length distribution of active phages entering bacterial genomes via lysogeny. The parameter r_D_ is the rate at which prophage sequences decay in length over time. Thus, before the action of selection and induction, mutation acts at a constant and uniform rate across the prophage genome, and mutation is deletion-biased, gradually eroding prophages. The parameter r_S_ is the selection coefficient (benefit or detriment) conferred to the host by an intact prophage, while S(x) is the fraction of this selective effect conferred by a prophage of length x. The parameter r_I_ represents the rate of induction, while I(x) is the probability that a prophage of length x may be lost by induction. I(x) itself depends on the parameter n_I_, which is the number of genes required for the loss of the prophage to occur via induction, ie. the number of genes necessary for the prophage sequence to excise from the host genome. The function δ(t) is a normalizing constant which ensures that the density integrates to one at all times. In the steady-state solution, this function is a constant which we simply denote δ, reflecting the turnover rate of the prophage population.

As described in Pattenden et al. [16], we used maximum likelihood minimization to find parameter values such that the steady state solution of Eq. (1) best fits the prophage length distribution in our datasets. We note that the steady state solution does not depend independently on all of the rates in the model but only on the ratio of these rates. In other words, only four of the five rate constants in the model are identifiable, and the output of data fitting is not the rates themselves, but their relative ratios. We used the degradation rate as the normalizing factor for this ratio, since the rate of degradation, a mutational rate, is not expected to differ between host classes. This data fitting procedure thus yields four relative rates, α/r_D_, r_S_/r_D_, r_I_/r_D_ and δ/r_D_, which correspond to: the relative rate of lysogeny (i.e. rate at which new prophages enter the genome), the selective effect (i.e. selective benefit to the host of carrying an intact prophage), the induction rate (rate at which fully competent prophages induce the lytic phase), and the turnover rate (loss of prophages from the population, independent of their length). This data fitting procedure also estimated n_I_, the number of genes required for induction.

Finally, we used a separate bootstrapping procedure to determine whether these five best-fit parameter values differed significantly between AAB and NAAB. Here, we pooled all the prophage sequence lengths from AAB and NAAB, drew samples from this pooled set with replacement, and computed the best-fit parameter values for each sample. We then compared whether the best-fit parameter values obtained for AAB or NAAB data differed significantly from the distribution of parameter values estimated from the pooled data. For each of AAB and NAAB, we drew 1000 samples from the pooled dataset, where in each case the number of prophage lengths in the sample was the same as the number in the AAB or NAAB datasets, respectively.

## Results

A total of 1860 prophages distributed in the order Hyphomicrobiales were found, 695 (37.4%) in AAB (*n* = 2; *Bartonella* and *Brucella*)*,* and 1165 (62.6%) in the NAAB genera (*n* = 56). The number of prophages found per genome in both cases was similar: 695 prophages in 223 genomes = 3.1 prophages/genome in AAB, compared with 1165 prophages in 337 genomes = 3.4 prophages/genomes in the NAAB genera of the order. The outcomes of PHASTER analysis in AAB showed 493 incomplete, 62 questionable and 140 intact prophages. The analysis of the NAAB prophages showed 667 incomplete, 224 questionable and 274 intact prophages (Fig. 1). The AAB genomes contained a significantly higher frequency of incomplete prophages, while a higher fraction of prophages was classified as questionable in the NAAB genomes.

**Fig. 2:**
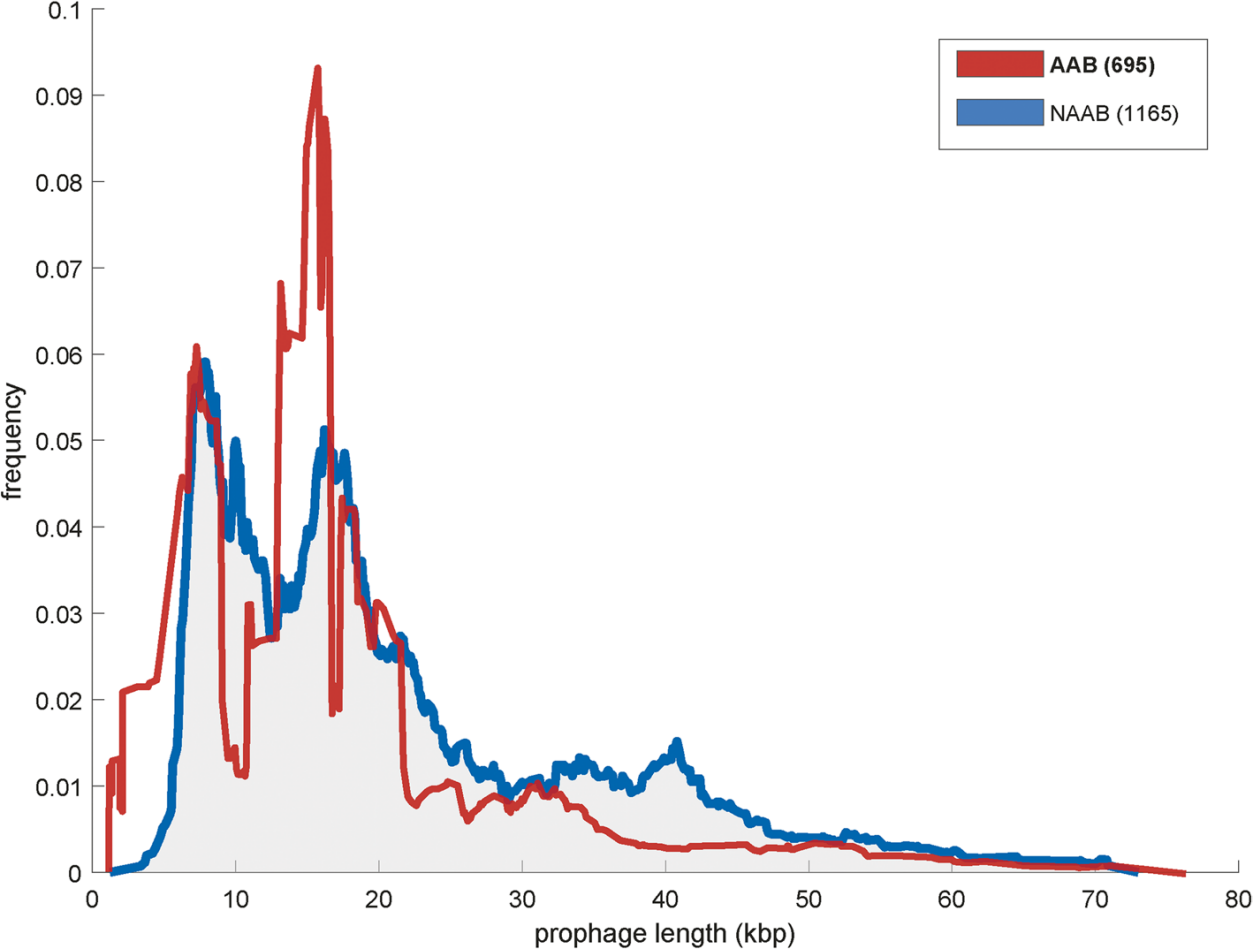
Length distributions of prophage sequences in host genomes: (blue) non-animal-associated bacteria (NAAB) and (red) animal-associated bacteria (AAB)

Turning to the prophage length distribution, we found that the AAB showed a high frequency of short prophage fragments between 5-25 kb with one peak at 5kbp and a second prominent peak at 15kbp (Fig. 2). The NAAB genomes also showed a high frequency of short prophages, however an additional frequency peak of long prophage sequences was found around 30-50 kb, such that this dataset exhibits a multimodal distribution of prophage lengths.

**Fig. 3:**
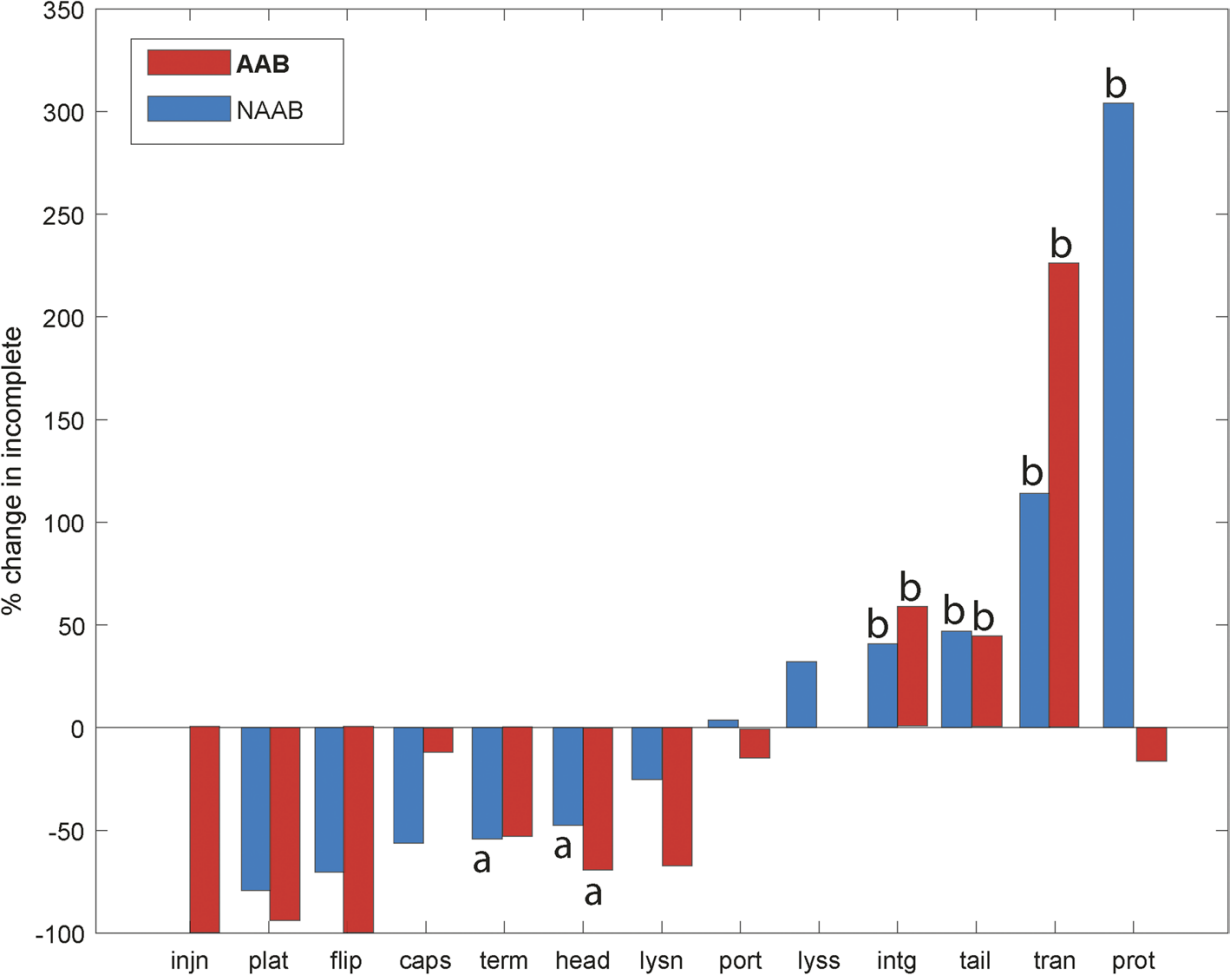
The percent change in gene frequency for incomplete prophages in animal-associated bacterial (AAB) and non-animal-associated bacteria (NAAB). Two categories of statistical significance were defined: **a** genes that are preferentially lost in incomplete prophages (**b**) genes enriched in incomplete prophages. Prophage gene repertoire: *injn*, injection; *plat*, plate; *flip*, flippase; *caps*, capsid; *term*, terminase; *head*, head; *lysn*, lysin; *port*, portal; *lyss*, lysis; *intg*, integrase; *tail*, tail; *tran*, transposase; *prot*, protease. Missing bars indicate that a gene class was not observed in intact prophages in the dataset, and thus the percent change is undefined

The percent change in the frequency of protein-coding genes, for each type of phage protein/enzyme, was compared between incomplete prophages versus intact prophages (Fig. 3). Two gene classes (injection genes in prophages of NAAB and lysis genes in prophages of AAB) were not observed in intact prophages in this dataset and were excluded from further analysis.

Using bootstrapped data to assess the significance of these changes, we found that incomplete prophages in AAB had preferentially lost one gene class (*head*), while three genes were enriched (*intg*, *tail*, and *tran*). The genetic repertoire of NAAB incomplete prophages showed that two genes were preferentially lost (*term* and *head*) and four genes were enriched (*intg*, *tail*, *tran* and *prot*). We also note the *y-*scale of Fig. 3: in NAAB for example, the frequency of protease genes in incomplete prophage sequences is three times higher than their observed frequency in intact prophage sequences.

**Fig. 4:**
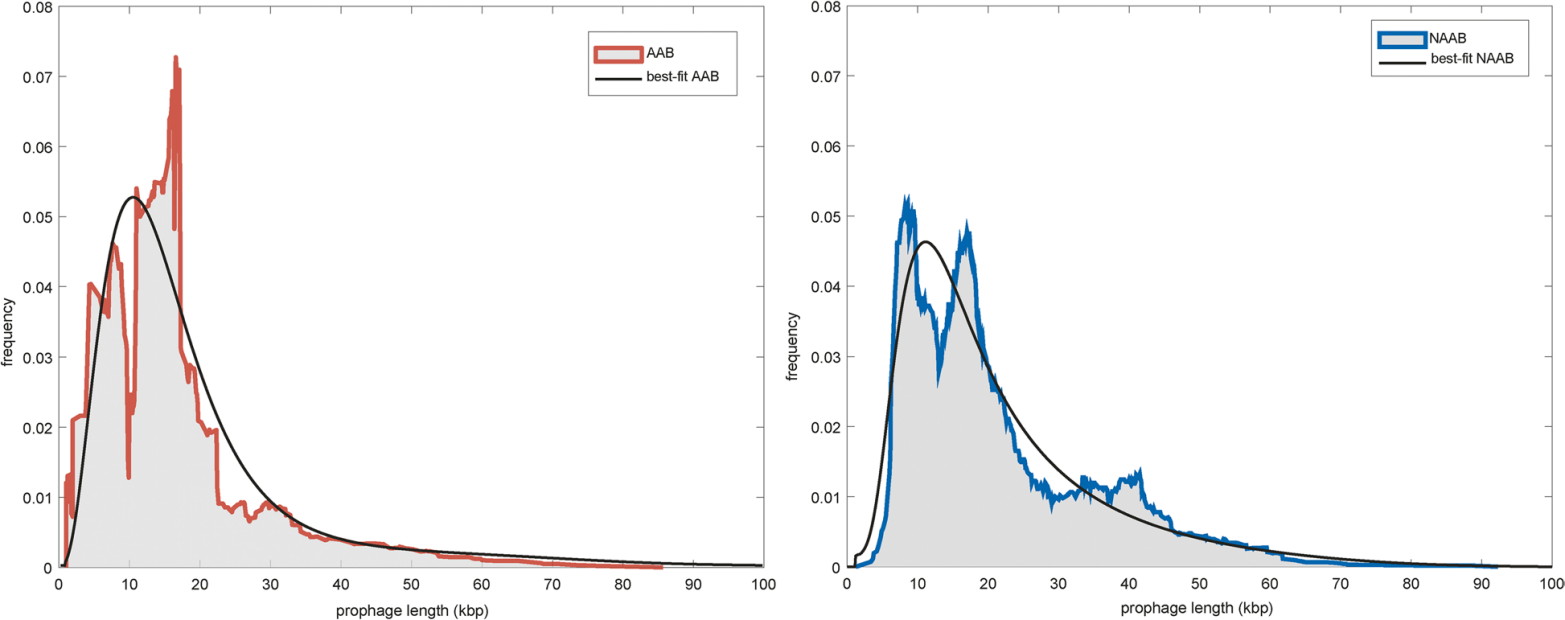
Data and best-fit prophage length distributions (black line) for (left/red) animal-associated bacteria (AAB) and (right/blue) non-animal-associated bacteria (NAAB)

**Fig. 5:**
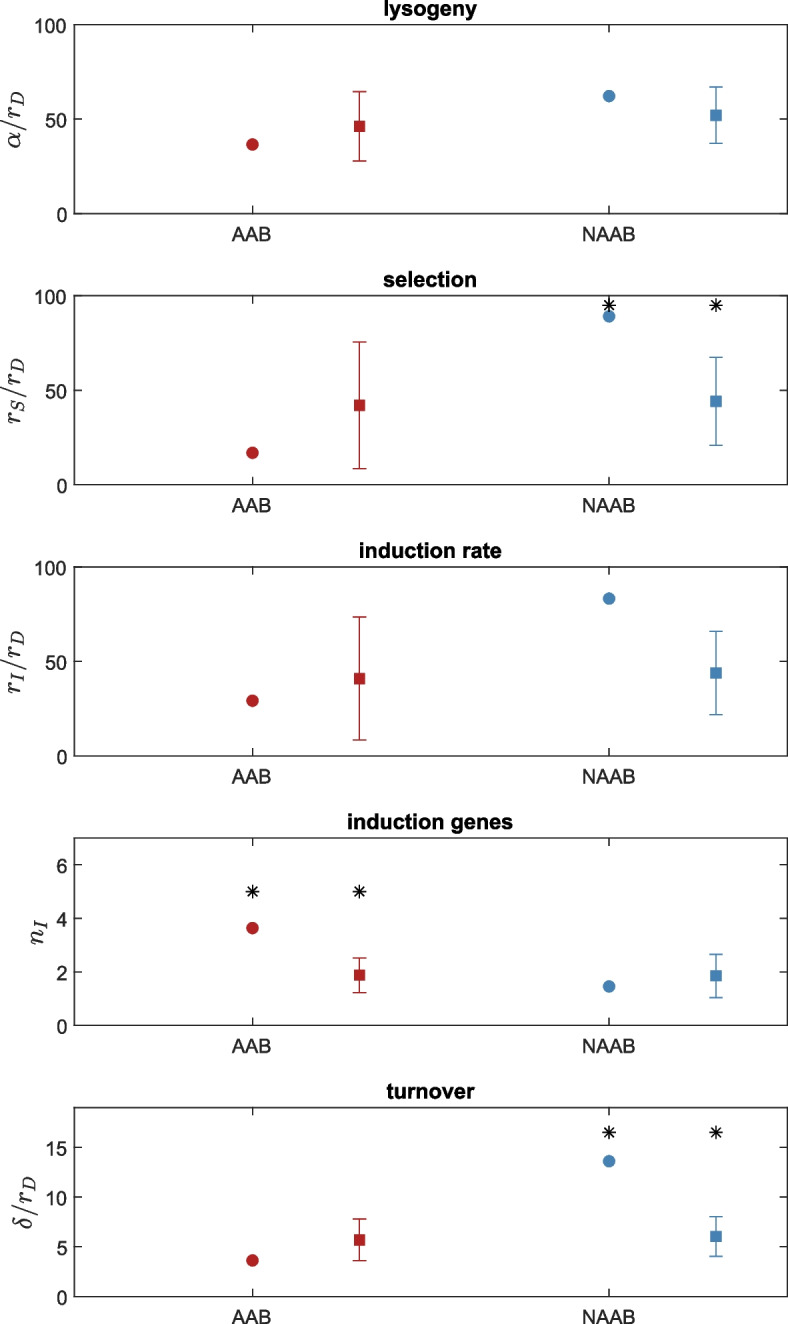
Sensitivity analysis: best-fit parameter values in the full datasets (circles) and in 1000 bootstraps of the pooled dataset (squares); parameters as indicated on y-axis. While the pooled dataset includes both AAB and NAAB, the size of each bootstrap sample was set to the number of prophages in the original dataset in each case, thus the error bars on the bootstrap results differ between AAB and NAAB. The stars indicate significant differences between the two groups

The best-fits of the stationary distribution of prophage lengths to the prophage length distributions in the data are illustrated in Fig. 4. The model captures the overall qualitative features of the distribution but does not capture the bi- or multi-modality of these distributions (see Discussion below and also Khan et al. [16]). Table 1 provides the best fit parameter values. While the numerical values of the parameters appear quite different between AAB and NAAB, these differences can only be interpreted in the context of the bootstrap sensitivity tests (Fig. 5). For example, the relative rate of lysogeny, α/r_D_, appears to be nearly twice as high in NAAB as in AAB. The sensitivity analysis, however, reveals that neither is significantly different from estimates obtained from the pooled dataset. Significant differences, however, were observed for three of the remaining four parameters. In particular, prophages in NAAB genomes show significantly higher selection coefficients for beneficial genes and turnover of the prophage population, while prophages in AAB genomes require significantly more genes to initiate induction. In addition, the data suggest that NAAB prophages experience higher induction rates, although this difference did not reach significance after the Bonferroni correction (Fig. 5). We also note that the selection coefficient in the best-fit model can be positive, indicating that before induction, intact prophages confer a net benefit to their host, or negative, indicating that intact prophages reduce host fitness. For both AAB and NAAB, the best-fit selection parameter was positive and significantly different from zero.

**Table 1:** Parameter values for the best fits for bacterial genomes for each class. Note that the rate parameters are relative rates and are thus unitless, while “induction genes” are in units of “number of genes”

Parameter	Animal-associated bacteria	Non-animal associated bacteria
**Lysogeny**	36.5	61.9
**Selection**	16.9	89.0
**Induction**	29.2	83.1
**Induction genes**	3.6	1.45
**Turnover**	3.6	13.6

We identified a total of 68 distinct types of APS within the order Hyphomicrobiales (see Supplementary Table 2). The number of APS found per genome in both cases was almost similar: 19 APS in 26 genomes = 0.7 APS/genome in AAB, compared with 67 APS in 70 genomes = 0.95 APS/genomes in the NAAB genera of the order. Among these systems, the restriction modification (RM) and abortive infection (Abi) mechanisms were the most frequently observed APS. In the case of the AAB group, consisting of 26 analyzed genomes, RM and Abi were again the predominant APS; however, it is noteworthy that certain strains of *Bartonella* exhibited either no APS or only possessed the RM and Abi systems. In contrast, within the *Brucella* genus, aside from these two predominant systems, some strains retained an additional 17 different APS, bringing the total count of identified APS to 19 (see Supplementary Table 2). For the NAAB group, analysis of 70 genomes revealed a remarkable diversity of APS, with 49 APS being exclusive to this group, expanding the overall understanding of APS in this context.

## Discussion

Our analyses indicate that the distribution of prophage length is comparable between the two datasets, implying broadly similar dynamics for prophage genes in both AAB and NAAB. These findings suggest that the processes underlying prophage integration and adaptation are consistent across these bacterial groups, potentially reflecting shared mechanisms of genetic acquisition and evolutionary dynamics.

Consistent with the results described by Bobay et al. [5], our results suggest that prophages of Hyphomicrobiales are under positive selection. This effect is significantly higher in NAAB, where despite a higher rate of induction (which means activation of the lytic cycle), there is significant selection for genes that benefit the host (Fig. 5). This could be related to ecological competition and a constant co-evolution process between these viruses and NAAB bacteria, consistent with the presence of several defense mechanisms in these genera [4]. The significantly higher rate of turnover and higher rate of induction observed for prophages in NAAB as opposed to the pooled data implies that prophages, in general, are less stably associated with NAAB genomes than with AAB genomes.

For most model parameters, the best-fit parameters predicted for AAB were not significantly different from parameter values in the pooled dataset. This could be explained by the lack of information on this group in the public databases, mainly for *Bartonella.* Additionally, our data suggest that in *Brucella* and *Bartonella* (AAB) genomes, more genes are required for induction. This indicates that the lytic phase may not be easily activated, suggesting a process of domestication of these prophages in the AAB genomes, a phenomenon that is typically expected for slow-growing bacteria [7].

Injection genes are a class of prophage genes that were not observed in NAAB while the lysis genes (*lyss*) were absent in AAB genomes (Fig. 3). The injection genes (*injn*) are related to the capability of a phage to transfer mobile elements into the hosting bacteria. The absence of this phage gene class is a unique trait of NAAB genomes. The absence of lysis genes (*lysis*) in incomplete prophages in AAB genomes could indicate that domestication (loss of lysis ability) is common in these genomes but could also be misidentification of lysis genes in these particular viral genomes. In Pattenden et al. [17], a significant enrichment of lysis genes was observed in slow-growing bacteria, while prophages in pathogenic bacteria showed a significant loss of lysis genes. *Bartonella* and *Brucella* are two genera of slow-growing bacteria, but most of the species in these two genera are classified as animal pathogens. Further detailed sequence analysis is required to resolve whether lysis genes are truly absent in many prophages in these genera, or whether such genes are rapidly lost due to sequence degradation or potentially due to active bacterial immune processes. In particular, due to the complexity of our bioinformatics pipeline, we used a single prophage screening tool in this analysis. A previous work found only minor differences when results obtained using PHASTER were compared with results obtained using the PATRIC database [13], however a range of prophage screening tools are now available and may differ in their ability to identify particular prophage genes.

The terminase genes (*term*) were preferentially lost in NAAB genomes, and the head genes in both datasets, in NAAB and AAB genomes (Fig. 3). Terminase genes encode the packing of phage DNA for assembling between capsid, portal and tail products to create a lytic phage, responsible for the translocation of phage DNA [18]. In both datasets the terminase genes were preferentially lost, suggesting a defense mechanism against the progeny of lytic phages. These latter results are consistent with previous studies of different datasets [16, 17], however this loss was only significant in NAAB genomes.

Integrase, tail and transposase (*intg*, *tail*, *tran*) genes are three classes of prophage genes that were shown to be enriched in prophage remnants in all genomes of Hyphomicrobiales (Fig. 3). These genes are expected to be enriched in incomplete prophages as observed in previous studies [16, 17]. Integrase genes are related with the attachment of phage genomes to the host bacterial genomes and are classified in tyrosine and serine integrase families [19, 20]. The high prevalence of these phage gene classes indicates the presence of pathogenic islands, and these regions also represent satellites of previous contacts between phages and host genomes [21, 22]. The presence of integrase genes in both datasets indicates that these genes are related with the integration sites present in Hyphomicrobiales genomes, and are selected in order to maintain the constant lysogenic process, as observed in *E. coli* and *S. enterica* [22]. The tail genes encode proteins to attach and infect bacterial cells, and are essential for their parasitic lytic cycle. They are also involved in the recognition, binding, and infection of bacterial cells. However, in different datasets tail genes were shown to be enriched in bacterial genomes and domesticated by hosting bacteria [16, 23, 24]. Tail genes could be related with increased phage resistance (like antibodies) to avoid the same phage infection [24], to increase the bacterial infectivity [23] or to deliver tailocins against other bacteria or even eukaryotic cells [25]. The transposase genes were also shown to be enriched in both datasets as shown in previous studies [16, 17]. This enrichment in short prophages, the most prevalent prophages found in our dataset, is possibly related to the disruption of intact phages by transposable elements [16]. Thus, the frequent appearance of transposase genes in incomplete prophage sequences may be the cause, rather than the result, of the transition from a prophage to a degraded, non-functional prophage sequence.

Proteases are genes significantly enriched in NAAB genomes, while they tend to be lost in AAB genomes (Fig. 3). Proteolysis is responsible for degradation of key proteins related to defense mechanisms against environmental changes like temperature and stress responses that could affect the bacteria [26]. Likewise, proteases play a key role in increasing the virulence of plant-associated bacteria [27]. Considering the lifestyle of NAAB (free living, nodule-associated or plant-associated; [6]), the presence of proteases acquired by phages could be advantageous against extracellular stress. These genes may be under weaker selection in the comparatively stable environment of AAB.

The enrichment of prophages is directly dependent on the defense mechanisms employed by bacteria to protect against phage infections, a relationship well-documented by Tesson et al. [12]. The presence of antiviral mechanisms is related to a number of factors, including lifestyle traits, viral threats, and genome size. In our study, we observed that the RM system, which is widely prevalent among prokaryotes, also played a significant role within the Hyphomicrobiales order. Since RM systems can directly contribute to DNA degradation [12], this finding may be linked to the abundance of short prophages in both groups. Specifically for the AAB group, we noted a substantial impact of lifestyle on gene loss, which in turn influenced genome size. While one might speculate that lysogeny could be a significant strategy within this group due to its relatively limited antiviral arsenal, our dataset did not provide concrete evidence to support this hypothesis. Notably, particularly in the case of *Bartonella*, the combination of a short genome size and the loss of anti-phage system (APS) genes in some species suggests the potential existence of widespread prophage domestication, or alternative protective mechanisms such as the presence of unidentified antiviral systems within this group. Conversely, in the NAAB group, despite the considerable diversity of APS identified, our findings indicated a pattern of high selection and turnover of prophages. This dynamic suggests a complex interplay between bacteria and their associated prophages within this group.

## Conclusion

Our study reveals an enrichment of incomplete prophages in the AAB genera compared to NAAB. Furthermore, it indicates that selection for beneficial prophage genes takes place in both AAB and NAAB. Additionally, our data implies that prophages exhibit greater stability and stronger associations with the genomes of AAB than NAAB. These findings shed light on these bacterial genera and emphasize the importance of prophages in shaping their genomic evolution. Further investigation of the functional implications of prophages in AAB will surely uncover valuable insights into their adaptation and survival strategies in the face of complex ecological constraints.

### Supplementary Information

**Additional file 1: Supplementary Figure 1.** Workflow of data mining and analyses of Order Hyphomicrobiales from BV-BRC/PATRIC dataset.

**Additional file 2: Supplementary Table 1.** Dataset used for PHASTER analysis of 560 genomes of Hyphomicrobiales. a Datasheet called “*BVBRC_genome*” with data of all genomes. b Datasheet called“*Contigs_PHASTER analysis*” with all the RefSeq numbers used for prophages analysis.

**Additional file 3: Supplementary Table 2.** Dataset used for DefenseFinder analysis of 96 genomes of Hyphomicrobiales. a Datasheet called “*database*” with data of all genomes and the defense systems found by DefenseFinder. b Datasheet called “*Heatmap*” with visualization of the defense systems separated by AAB and NAAB. c Datasheet called“*freq_DefenseFinder*” the frequence of the occurrence of each APS in Hyphomicrobiales.

## Data Availability

The data that support the findings of the current study are available at the Supplementary data (Fig. 1 and Table 1a, b and Table 2a-c) and from the corresponding author upon reasonable request. The Hyphomicrobiales genome sequences analyzed in this study are available in Bacterial and Viral Bioinformatics Resource Center (https://www.bv-brc.org/).
