# Supplementary material for: Exploring the diversity and evolutionary strategies of prophages in Hyphomicrobiales, comparing animal-associated with non-animal-associated bacteria
Source: BMC Microbiol. 2024 May 9;24:159. doi: 10.1186/s12866-024-03315-3 (PMC11080155; doi:10.1186/s12866-024-03315-3)

Supplementary Figure 1 – Workflow of data mining and analyses of Order Hyphomicrobiales from BV-BRC/PATRIC dataset

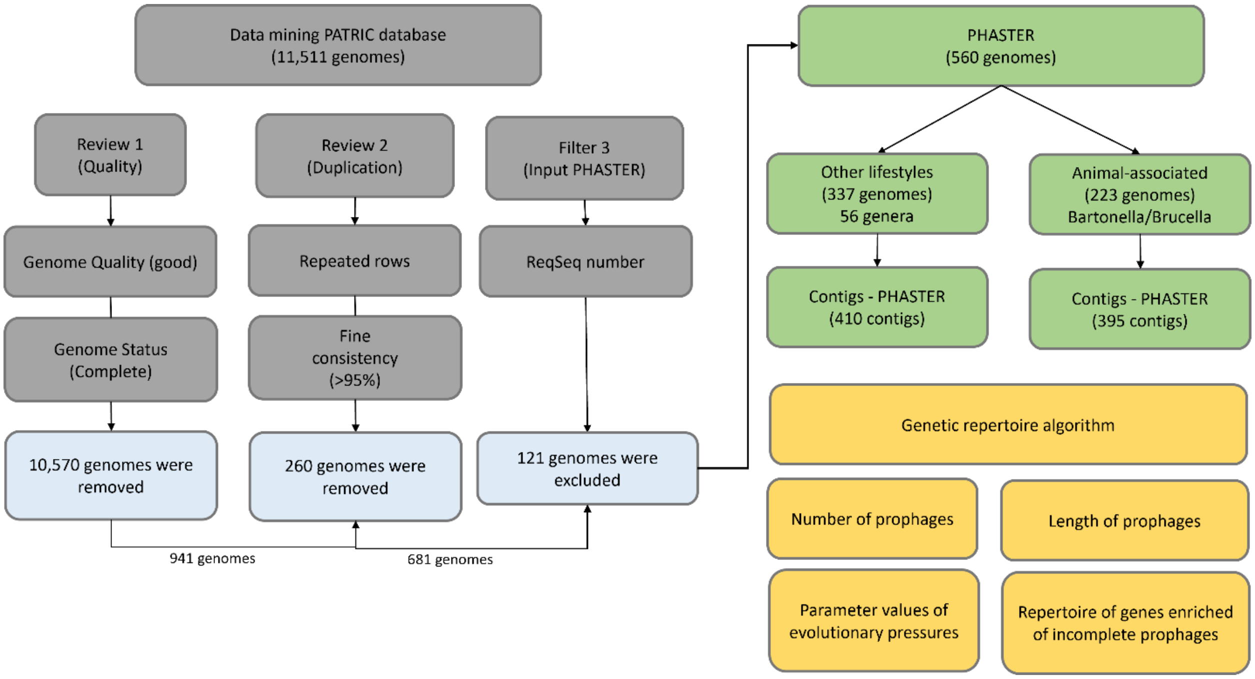

Supplement: Supplementary file 1 — Additional file 1: Supplementary Figure 1. Workflow of data mining and analyses of Order Hyphomicrobiales from BV-BRC/PATRIC dataset. [file 12866_2024_3315_MOESM1_ESM.pdf]
